# Fluid Redistribution in Sleep Apnea: Therapeutic Implications in Edematous States

**DOI:** 10.3389/fmed.2017.00256

**Published:** 2018-01-22

**Authors:** Bruno Caldin da Silva, Takatoshi Kasai, Fernando Morgadinho Coelho, Roberto Zatz, Rosilene M. Elias

**Affiliations:** ^1^Renal Division, Department of Medicine, Universidade de São Paulo, São Paulo, Brazil; ^2^Cardiovascular Respiratory Sleep Medicine, Department of Cardiology, Juntendo University Graduate School of Medicine, Tokyo, Japan; ^3^Department of Neurology, Universidade Federal de São Paulo, São Paulo, Brazil; ^4^Department of Psychobiology, Universidade Federal de São Paulo, São Paulo, Brazil

**Keywords:** sleep apnea, fluid overload, edema, fluid shift, continuous positive airway pressure, congestive heart failure, chronic kidney disease

## Abstract

Sleep apnea (SA), a condition associated with increased cardiovascular risk, has been traditionally associated with obesity and aging. However, in patients with fluid-retaining states, such as congestive heart failure and end-stage renal disease, both prevalence and severity of SA are increased. Recently, fluid shift has been recognized to play an important role in the pathophysiology of SA, since the fluid retained in the legs during the day shifts rostrally while recumbent, leading to edema of upper airways. Such simple physics, observed even in healthy individuals, has great impact in patients with fluid overload. Correction of the excess fluid volume has risen as a potential target therapy to improve SA, by attenuation of nocturnal fluid shift. Such strategy has gained special attention, since the standard treatment for SA, the positive airway pressure, has low compliance rates among its users and has failed to reduce cardiovascular outcomes. This review focuses on the pathophysiology of edema and fluid shift, and summarizes the most relevant findings of studies that investigated the impact of treating volume overload on SA. We aim to expand horizons in the treatment of SA by calling attention to a potentially reversible condition, which is commonly underestimated in clinical practice.

## Introduction

Sleep apnea (SA) is a condition characterized by repeated episodes of complete or partial airflow cessation during sleep, typically referred as apnea and hypopnea. Individuals with SA usually present witnessed episodes of snoring, choking, and are more likely to suffer from daytime sleepiness (1), depression (2, 3) and are at increased risk of motor vehicle crash (4), and occupational accidents (5). Other important adverse consequences of SA include neuropsychiatric disorders, such as cognitive impairment (6), abnormal sympathetic activity (7), and cardiovascular abnormalities such as hypertension (8), stroke, and arterial obstruction (9).

The apnea–hypopnea index (AHI), defined as the total number of episodes of apnea and hypopnea per hour of sleep, is routinely used to diagnose SA and to classify it as mild (AHI between 5 and 15), moderate (15–30), or severe (>30) (10). The prevalence of AHI > 5 is 9% in women and 24% in men in the general population (1), not taking into account the presence of symptoms. Nevertheless, the prevalence of SA increases over time, since obesity, one of the most important risk factors, has increased in general population. More recent data suggest that more than 20% of adults have mild SA and up to 7% have moderate or severe SA (11).

Even though aging and obesity are clearly the most relevant associated risk factors, the prevalence of SA is much higher among patients with edematous states, such as end-stage renal disease (ESRD) (12) and congestive heart failure (CHF) (13). Hypervolemia and overnight rostral fluid shift from the legs are the likely cause of the high frequency of SA in edematous states, as indicated by several recent studies of ESRD (14), CHF (13), and nephrotic syndrome (15) (Table 1).

**Table 1:** Studies on the relationship between volume overload and SA that have included patients with fluid-retaining states.

Study	Condition	Population	Methods	Findings
Inoshita et al. (39)	CHF	17 patients with CHF vs. 34 without CHF matched for BMI and OSA severity	Craniofacial anatomy evaluation	Patients with CHF had larger, edematous tongue and more collapsible airway
Kasai et al. (41)	CHF	18 patients with obstructive and 10 central-dominant SA	LBPP by using anti-shock trousers for 15 min	LBPP reduced LFV and increased NC. Transpharyngeal resistance and PCO_2_ increased in patients with OSA, while the opposite occured in CSA-dominant patients
Yumino et al. (13)	CHF	57 patients with obstructive or central-dominant SA	BIS, PSG, and overnight NC variation	Reduction in LFV correlated inversely with AHI and overnight change in NC in all patients and also correlated positively with PCO_2_, only in patients with CSA
Kasai et al. (42)	CHF	35 men and 30 women with CHF	BIS, PSG, and overnight NC variation	Overnight NC variation was lower in women, despite the same fluid displaced from the legs. AHI severity was significantly correlated with fluid shift in men but not in women
Elias et al. (14)	ESRD	26 patients on HD	BIS, PSG, and overnight NC variation	Change in LFV was inversely correlated with apnea-hypopnea time and change in overnight NC
Lyons et al. (61)	ESRD	21 patients on HD	BIS, PSG, and echocardiogram	In men, AHI correlated with left atrial size, while LFV variation correlated with AHI and left atrial size
Elias et al. (60)	ESRD	20 patients on HD	BIS, PSG, and MRI	Increased upper airway water content and internal jugular vein volume were positively correlated with AHI
Lyons et al. (62)	ESRD	15 patients on HD	BIS, PSG	A single ultrafiltration session (2.17 ± 0.45 L) decreased AHI by 36%
Tang et al. (15)	Nephrotic syndrome	23 patients with nephrotic syndrome and lower limb edema	BIS, PSG	Reduction in extracellular body water after nephrotic syndrome treatment attenuated SA from 16.3 ± 5.1 to 7.8 ± 2.3 events/h

*CHF, congestive heart failure; pts, patients; BMI, body mass index; OSA, obstructive sleep apnea; SA, sleep apnea; LBPP, lower body positive pressure; LVF, leg fluid volume; NC, neck circumference; CSA, central sleep apnea; BIS, bioimpedance spectroscopy; PSG, polysomnography; AHI, apnea–hypopnea index; MRI, magnetic resonance imaging; HD, hemodialysis; ESRD, end-stage renal disease*.

Despite its high prevalence in edematous patients, SA is often overlooked because of its oligosymptomatic nature (16, 17). Even when SA is adequately diagnosed by polysomnography, management of this condition is of great concern, since the gold standard treatment, the use of continuous positive airway pressure (CPAP) (18–21), presents low compliance rates (22, 23).

Although prevention of fluid accumulation is a plausible alternative strategy to alleviate SA in edematous patients, current guidelines do not include treatment of edema as part of the therapeutic effort against this condition. In this review, we discuss the impact of edema on the pathogenesis of SA in patients with CHF, ESRD and nephrotic syndrome, as well as the corresponding implications for innovative therapeutic strategies.

## Pathophysiology of Edema: A Summary

### Edema: Interstitial Accumulation of Sodium and Water Retained by the Kidneys

Edema is defined as an abnormal buildup of fluid anywhere in the body. When utilized with no qualifier, the term “edema” usually refers to the accumulation of plasma transudate in the interstitial space, as in CHF, nephrotic syndrome, and hepatic cirrhosis (24–26).

For fluid to accumulate at the interstitial space, a positive sodium balance must establish. Since the kidneys are ultimately responsible for maintaining sodium balance, it follows that edema formation always demands some degree of renal sodium retention. Nevertheless, impaired sodium excretion is insufficient to ensure fluid accumulation. For instance, in primary hyperaldosteronism, excess sodium reabsorption by the distal nephron translates into hypertension, rather than edema formation. To reach the interstitial space, fluid retained by the kidneys must be driven by an imbalance of Starling forces at the complex interface between the intravascular and interstitial compartments (27).

Under normal conditions, small amounts of fluid do reach the interstitial compartment due to a slight predominance of hydrostatic over oncotic forces. Actually, the normal interstitium contains about 10 L of fluid, an amount kept within narrow limits by three mechanisms (27): the action of lymphatic capillaries, carrying extravasated fluid back to the circulation; the dilution of interstitial protein that results from transcapillary fluid passage; and the tight disposition of the protein molecules that constitute the interstitial matrix—due to this arrangement, substantial elevation of local hydraulic pressure is required to accommodate even small amounts of extra fluid (low interstitial compliance).

An important consequence of these physical characteristics of the normal interstitial matrix is that fluid cannot move freely across the interstitium following gravity and, therefore, will not accumulate in the lower limbs while standing, or in the cervical region after several hours in the recumbent position.

**Figure 1:**
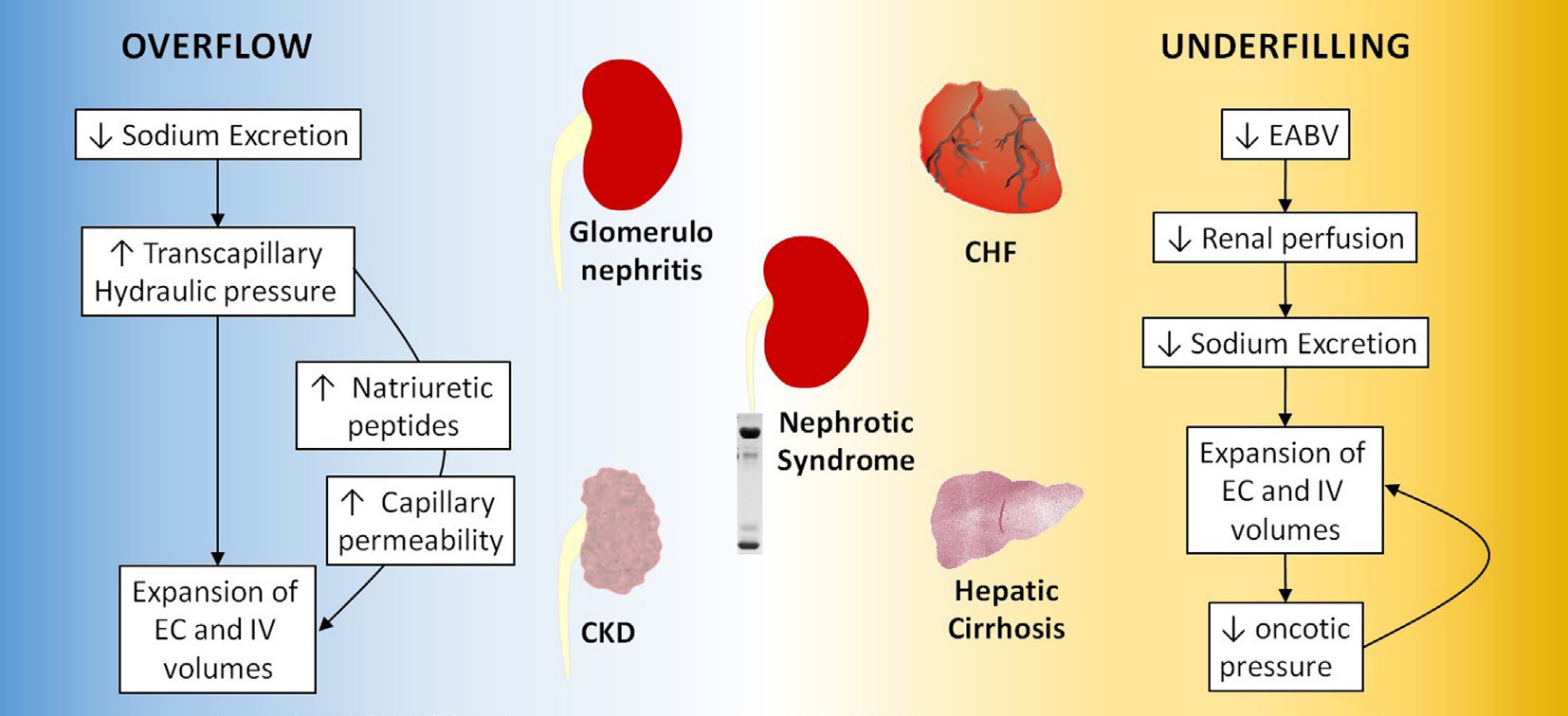
Overflow and underfilling mechanisms in edematous states. CKD, chronic kidney disease; EC, extracellular; IV, intravascular; CHF, congestive heart failure.

Although the classical view centered on Starling forces still predominates, recent evidence suggests that this theory should be revised taking into account the segmentation of the capillary wall and the adsorption of ions by interstitial macromolecules (28, 29). An overview of the mechanisms evolved in edema formation is summarized in Figure 1.

### Edema Primarily due to Impaired Renal Sodium Excretion: The Overflow Mechanism

When sodium excretion is hindered by intrinsic renal disease, a positive sodium balance establishes, leading to expansion of the extracellular (EC) and intravascular volumes. If Starling equilibrium is not disrupted, sodium retention will cause hypertension, according to Guyton’s theory (27), but not edema. However, if tissue autoregulation fails, capillary hydraulic pressure will rise, and the resulting imbalance of Starling forces will lead to fluid extravasation. This mechanism of fluid retention, known as overflow (also overfill), operates in primary renal disease, such as glomerulonephritis (30) and advanced chronic kidney disease (CKD). As EC volume is expanded, mechanisms that increase sodium excretion are triggered, counteracting the renal limitation and allowing a new sodium balance to be reached. For this reason, edemas accumulated by overflow are relatively modest and confined to the lower limbs and eyelids.

In patients on chronic dialysis, maintenance of fluid balance is entirely dependent on an artificial procedure. Therefore, development of edema in this context usually results from insufficient fluid removal (31) or poor adherence to treatment. Thus, the mechanism of edema formation in these patients can be considered as analogous to overflow.

### Do Not Blame the Kidneys: Edema Formation due to Circulatory Underfilling

In a number of situations, effective arterial blood volume (EABV), hence renal perfusion, cannot be maintained despite normal or even increased total blood volume. In this context, the kidneys (assumed to be normal) react to the reduction of EABV by retaining sodium and water, which nevertheless escape the intravascular space because of a disequilibrium of Starling forces, promoting further sodium retention. In this manner, the retained fluid tends to accumulate at the interstitial compartment, instead of recomposing the EABV (32).

This mechanism of edema formation resulting from chronic reduction of the EABV is known as underfilling. Unlike what happens with overflow, here renal dysfunction is not the primary cause of sodium retention. Rather, the kidneys act as expected, responding to hypoperfusion by reabsorbing as much sodium as possible. Underfilling is central to the pathogenesis of edema in CHF, hepatic cirrhosis and some cases of nephrotic syndrome.

### Edema in CHF: Weak Pump, Low EABV, High Venous Pressure

Under normal conditions, the heart easily meets the needs of all tissues, keeping cardiac output at physiological levels. In CHF, the weakened myocardium can no longer maintain adequate perfusion of the peripheral territories, including the renal circulation. The consequent fall of EABV stimulates the kidneys to retain sodium. On the other hand, the malfunctioning pump leads to venous damming of blood. Retrograde transmission of the resulting venous hypertension to the capillaries promotes the passage of fluid to the interstitial space. This process is continuously fueled by the renal retention of sodium, which nevertheless fails to restore the EABV. Therefore, the basic mechanism of edema formation in CHF is underfillling (33).

### Formation of Edema in Nephrotic Syndrome: Underfilling or Overflow?

About one-third of patients with nephrotic syndrome exhibit clear signs of hypovolemia despite massive EC fluid expansion. In these patients, edema formation is believed to result from hypoalbuminemia, hence decreased systemic oncotic pressure, leading to an imbalance of starling forces, fluid displacement to the interstitium, EABV reduction, and incessant renal sodium retention. This sequence is fully compatible with the concept of underfilling (25, 34). However, two-thirds of nephrotic patients exhibit clear clinical evidence of fluid overload. It is believed that, in these patients, the basic event is primary sodium retention by the kidneys, with hypoalbuminemia facilitating ultrafiltration through the capillary walls, so that the magnitude of swelling is much higher than in the nephritic syndrome. Therefore, the basic mechanism of edema formation in most cases of nephrotic syndrome is overflow, facilitated by the simultaneous decrease in plasma oncotic pressure (2, 12).

### Edema Accumulation Deeply Changes the Physical Properties of the Interstitial Space

If fluid escape into the interstitium persists, the initially slow accumulation of edema raises gradually the local hydraulic pressure, until it becomes positive. When this happens, the normally tight architecture of the interstitium is disrupted, leading to an abrupt increase of compliance, enabling the interstitium to accommodate increasing amounts of fluid with a small rise of hydraulic pressure. The shift of fluid throughout the interstitium is no longer restricted, being now governed by gravity: during daytime, edema accumulates in the lower limbs; at night, interstitial fluid tends to be redistributed rostrally, reaching the cervical region. These movements largely explain the occurrence of airway obstruction and SA in edematous states.

Sleep apnea can be classified as obstructive sleep apnea (OSA), associated with airway obstruction and, therefore, respiratory effort, or central sleep apnea (CSA), in which the main pathogenic factor is respiratory center instability. The most common sleep disorder is OSA. CSA is far less common although equally as dangerous as OSA.

## SA in CHF

Both SA modalities are more prevalent in patients with CHF, compared to the general population (1, 35–37), especially in the case of CSA, which affects 21–40% of CHF patients, as compared to less than 1% of the general population (38). In CHF patients, fluid retention, and in particular fluid shift can cause not only upper airway obstruction by local fluid accumulation, but also pulmonary congestion. Despite their different pathogeneses, OSA and CSA can occur simultaneously in patients with CHF. Actually, fluid shift can participate in both SA types and fluid overload can explain the higher prevalence of both OSA and CSA in patients with CHF (13).

### Role of Edema and Fluid Shift in OSA in CHF

In CHF, fluids displaced from the lower body during the night can accumulate at cervical and head areas, thus promoting upper airway obstruction and OSA. It has been postulated that systemic fluid retention, with consequent venous engorgement and mucosal fluid accumulation, can increase tongue volume, facilitating airway obstruction (39). Of note, fluid accumulation in the neck, causing mucosal edema and OSA, was seen in healthy men after IV saline infusion during sleep (40).

In men with CHF and OSA, Kasai and colleagues showed that application of lower body positive pressure (LBPP) in the awake state, thus forcing rostral fluid shift, was accompanied by a significant increase in neck circumference and an increase in upper airway resistance in proportion to the volume of fluid displaced from the legs (41). Interestingly, the relationship between rostral fluid shift and OSA in CHF is less pronounced in women (13, 42).

### Role of Edema and Fluid Shift in CSA in CHF

In CHF patients, fluid retention and fluid shift from the legs can also lead to pulmonary congestion. In this case, however, SA is unrelated to obstruction. Rather, it seems to result from a central respiratory mechanism, thus conforming to the CSA type.

The mechanism underlying the establishment of CSA in these patients has not been fully elucidated, although pulmonary congestion, increased central and peripheral chemosensitivity, and frequent arousals may play a role (43, 44). Pulmonary congestion, a common finding in CHF, can stimulate so-called pulmonary vagal irritant “J” receptors (45), causing reflex inhibition of the respiratory drive through afferent C fibers. The consequent apnea causes PaCO_2_ to increase, now leading to hyperventilation and generating a Cheynes–Stokes-like pattern (35, 37, 46). In consistency with this concept, PaCO_2_ in CHF is inversely proportional to pulmonary capillary wedge pressure (47), which is an index of pulmonary congestion (48).

### Salt Intake

Dietary sodium intake can be associated with the severity of both OSA and CSA in CHF patients, with increased sodium intake presumably resulting in worsening of edema around the upper airway (OSA) and/or pulmonary congestion and CSA, through the mechanisms discussed earlier. Increased leg fluid retention, and consequently nocturnal overnight rostral fluid shift, can also be favored by excessive sodium intake (49).

## SA in Kidney Disease

The presentation of SA in patients with ESRD is quite distinct from that in the general population. First, the typical history of loud snoring and witnessed apnea during sleep is seldom obtained. Second, the association with age, gender, and body mass index is less clearcut (50). Third, even classical symptoms such as daytime sleepiness are infrequent and dissociated from the severity of SA (51). Together, these atypical clinical characteristics can render the diagnosis of SA quite difficult in ESRD patients.

Sleep apnea can exert a high impact on CKD mortality (52), given is very high prevalence (up to 80%) among these patients (14, 53, 54), and its well-known association with cardiovascular events (8, 55, 56). Therefore, recognizing SA in this population is imperative.

Uremia has been implicated as a possible cause of SA (57, 58). This concept, based on anecdotal reports of symptom improvement following renal transplantation, have been disputed (59). It must be noted that, even if these observations were confirmed by large clinical trials, interpretation would be problematic, given the plethora of factors that can be ameliorated after kidney transplantation.

### Role of Edema and Fluid Shift in SA in Kidney Disease

Rostral fluid shift may exert a similar influence in CKD as in CHF. In a study of 26 patients on conventional hemodialysis, Elias and coworkers (14) showed that SA, present in 46.1% of subjects, was associated with age, male gender, and time spent in the sitting position during the day. Rostral fluid shift correlated significantly with the severity of SA and with the overnight increase of neck circumference. In a related study, fluid shift was shown to correlate with the increase of internal jugular vein volume, mucosal water content, and AHI (60). Likewise, Lyons et al. (61) showed a correlation between the magnitude of rostral fluid shift and the severity of both OSA and left atrial size in 40 patients on conventional hemodialysis, reinforcing the view that fluid shift may have an impact on both OSA and cardiac dysfunction in ESRD. The proof of concept that fluid overload can impact in the severity of OSA in patient on dialysis was demonstrated by Lyons and coworkers, who showed that AHI fell by 36% after removal of an average of 2.2 L by ultrafiltration alone in ESRD patients (62).

The importance of fluid retention in the pathogenesis of SA is not restricted to CKD and ESRD. In patients with nephrotic syndrome, even with normal renal function, the treatment of hypervolemia, with contraction of EC volume and disappearance of lower limb edema, was shown to alleviate SA (15).

In summary, patients with kidney disease, particularly those on dialysis, and patients with nephrotic syndrome are more prone to have SA. The role of fluid overload and overnight fluid shift as risk factors for SA were well demonstrated in these settings (14, 15, 54, 60, 62–66). The data presented in these cited studies suggest that kidney disease and nephrotic syndrome might cause SA independently of confounding factors. Fluid overload *per se* contributed to the presence of SA in patients on hemodialysis that can be partly reversible through fluid removal by ultrafiltration (67).

## Targeted Therapy of SA in Fluid-Retaining States

**Figure 2:**
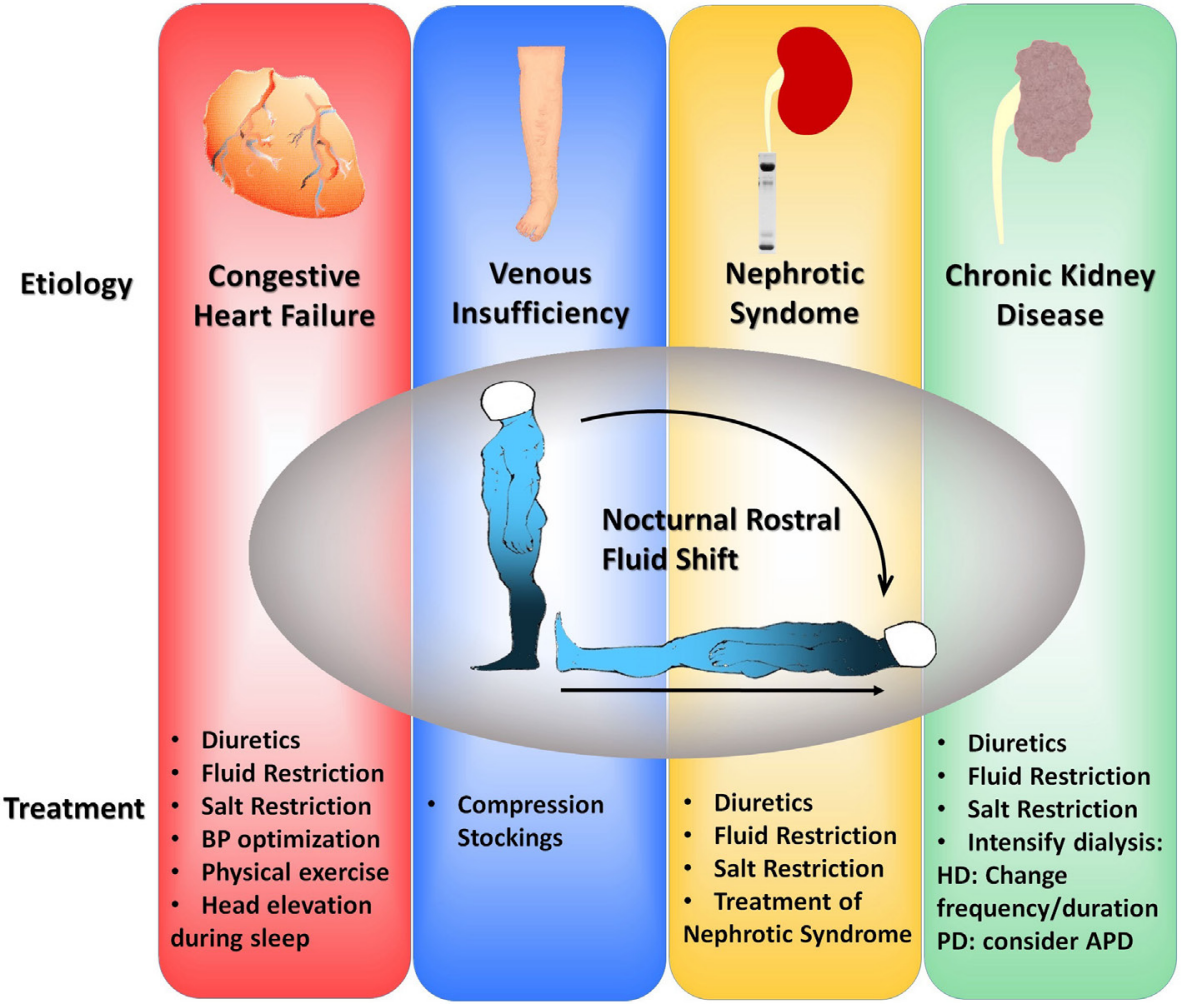
Flowchart of suggested therapeutic interventions to alleviate fluid shift in four different clinical scenarios: congestive heart failure, venous insufficiency, nephrotic syndrome, and chronic kidney disease. BP, blood pressure; HD, hemodialysis; PD, peritoneal dialysis; APD, automatic peritoneal dialysis.

Below, we describe different treatment options for the management of OSA. All these treatments are summarized in Figure 2.

### Continuous Positive Airway Pressure

Basically, the therapeutic action of CPAP is to mechanically impede the collapse of the upper airways, thus preventing OSA. In addition, CPAP prevents CSA because it maintains a continuous airflow. CPAP seems to have no effect on overnight fluid shift in patients on hemodialysis (68), although is considered the mainstream treatment for OSA regardless of volume overload given its mechanism of action.

### Diuretics, Fluid, and Salt Restriction

Targeted therapy for fluid retention and/or rostral fluid shift has been tested in several studies. In CHF patients with left ventricular diastolic dysfunction and severe OSA, intensive diuretic therapy increased upper airway cross-sectional area and lowered AHI by 24% (69). Increased physical activity during cardiac rehabilitation has also been associated with attenuation of both OSA and CSA (70, 71), possibly by preventing lower body fluid accumulation.

### Head-Elevated Patient Positioning

Head-elevated patient positioning can ameliorate OSA in CHF by preventing cervical fluid accumulation (72). Interestingly, this maneuver can also prevent CSA in CHF patients. Similar results were obtained in CHF patients with predominant OSA (72), with no effect on thoracic fluid content or left ventricular hemodynamics (73). This effect was attributed to increased venous return and dilation of the left heart while in the supine position, although lung congestion may also play a role.

### Increase of Dialysis Dose

As remarked earlier in this review, overflow is the mechanism of fluid retention in patients on hemodialysis. Accordingly, amelioration of SA in this population is expected to be proportional to the efficiency of fluid removal. In patients on conventional hemodialysis (three 4-h sessions/week), the SA severity tends to increase during interdialytic periods, reaching a maximum immediately before each session (12, 16, 51). Therefore, increasing the duration and/or frequency of sessions, thus mimicking more faithfully the operation of normal kidneys, may be a sound strategy to prevent SA in ESRD. In a study of 14 patients transferred from conventional to intensive hemodialysis (five 6-h sessions/week), AHI decreased by 68%, with marked improvement of oxygen saturation (63). A similar trend was observed in patients treated with peritoneal dialysis (PD) (74). Tang et al. (65) showed that in patients undergoing automated nocturnal PD, in which fluid removal was more efficient than with the manual procedure, a greater reduction of AHI was achieved, in association with less airway obstruction.

### Treatment of the Nephrotic Syndrome

The importance of fluid retention is highlighted by the behavior of SA in patients with nephrotic syndrome, even when renal function is normal. In patients with steroid-responsive nephropathy, Tang et al. (15) showed that the severity of SA was reduced after kidney disease remission, in association with disappearance of lower limb edema and reductions in body water content.

### Compression Stockings

Reducing leg swelling by wearing compression stockings during the day attenuated SA in patients with venous insufficiency (75, 76) and ESRD (68). This beneficial effect was observed in a general OSA population (77), highlighting the impact that even small amounts of fluid retained in the legs during the day might have on SA.

Table 2 summarizes several studies in which the efficacy of the aforementioned therapies was tested.

**Table 2:** Studies that evaluated the impact of target therapies on SA in patients with fluid overload conditions.

Study	Condition	Population	Targeted Therapy	Findings
Bucca et al. (69)	Diastolic HF	15 patients with severe OSA	Furosemide + Spironolactone for 3 days	AHI reduced from 74.89 ± 6.98 to 57.17 ± 5.40 events/h, associated with reduced body weight, improvement of or pharyngeal junction area and respiratory flow
Yamamoto et al. (70)	CHF	10 patients included in cardiac rehabilitation program vs. 8 control patients	Aerobic exercise training for 6 months	AHI remained stable in control group after 6 months, from 30.4 (19.9; 36.3) to 36.6 (8.6; 39.4) and improved after training: from 24.9 (19.2; 37.1) to 8.8 (5.3; 10.1) events/h. CSA, but not OSA, improved
Ueno et al. (71)	CHF	8 patients with OSA, 9 with CSA and 7 without SA	Aerobic exercise training for 4 months	In patients with OSA, AHI was reduced in 36% after exercise training
Soll et al. (73)	CHF	25 patients with Cheyne–Stokes apneas or hypopneas (index > 5 events/h)	Changes in sleeping angle degrees	Moving patients from 0 to 45° reduced AHI from 34.7 ± 30 to 23.2 ± 23.7 events/h
Basoglu et al. (72)	CHF	30 patients with diagnosed OSA	Changes sleep angle from 0 to 45°	AHI reduced from 30.8 ± 20.7 to 17.8 ± 12.1 events/h
Hanly and Pierratos (63)	ESRD	14 patients with diagnosed SA	Switching from conventional (4 h, 3 times a week) to nocturnal HD (8 h, 6–7 times a week)	AHI reduced from 25 ± 25 to 8 ± 8 events/h
Tang et al. (65)	ESRD	24 incident dialysis patients	Performing nocturnal cycler-assisted peritoneal dialysis before initiating CAPD program	AHI increased from 3.4 ± 1.34 to 14.0 ± 3.46 events/h after starting CAPD. TBW was significantly lower comparing nocturnal cycler-assisted PD with CAPD (32.8 ± 7.37 vs. 35.1 ± 7.35 L)
Redolfi et al. (76)	Venous Insufficiency	12 patients with diagnosed SA	Compression stockings for 1 week	AHI reduced 36% after wearing compression stockings

*HF, heart failure; pts, patients; OSA, obstructive sleep apnea; AHI, apnea-hypopnea index; CSA, central sleep apnea; CHF, congestive heart failure; SA, sleep apnea; HD, hemodialysis; CAPD, continuous ambulatory peritoneal dialysis; TBW, total body water; PD, peritoneal dialysis; ESRD, end-stage renal disease*.

## Conclusion and Further Directions

In the search for alternatives to CPAP, it is imperative to understand the pathogenic role of fluid overload and overnight fluid shift. The association of CPAP with strategies aimed at limiting edema and rostral fluid shift may reduce the need for high airway pressure, thus improving tolerance. Further work is required in order to assess cardiovascular outcomes of treating SA by interference on fluid overload/redistribution. Additionally, CPAP alone has failed to improve mortality among patients with OSA. Nevertheless, it is unclear if adding a fluid restriction strategy would change such outcomes.

## Author Contributions

Concept and design: BS and RE; data interpretation: BS, TK, FC, RZ, and RE; manuscript writing: BS, TK, RZ, and RE; final approval of manuscript: BS, TK, FC, RZ, and RE.

## Conflict of Interest Statement

The authors declare that the research was conducted in the absence of any commercial or financial relationships that could be construed as a potential conflict of interest.

## Abbreviations

AHI, apnea–hypopnea index; CHF, congestive heart failure; CKD, chronic kidney disease; CPAP, continuous positive airway pressure; CSA, central sleep apnea; EABV, effective arterial blood volume; ESRD, end-stage renal disease; LBPP, lower body positive pressure; OSA, obstructive sleep apnea; PCWP, pulmonary capillary wedge pressure; SA, sleep apnea.
